# Retraction: Vimentin Is a Novel Anti-Cancer Therapeutic Target; Insights from *In Vitro* and *In Vivo* Mice Xenograft Studies

**DOI:** 10.1371/journal.pone.0214006

**Published:** 2019-03-21

**Authors:** The PLOS ONE Editors

Following publication of this [1] article, the following concerns were raised:

- Potential overlap in dot plot patterns within regions of the following pairs of figure panels: Fig 2A HT1080 24h/μ1M and SKLMS1 4h/5μM dot plot; Fig 3C VimKD DMSO and Fig 2A STS26T DMSO dot plot; Fig 4C KD+pcDNA DMSO and WFA (1uM/24h) dotplot;
- Duplication of patterns within the background of blots shown in Fig 4A (DMSO lane in both DMSO and Z-VAD blots), Fig 4C (lane 1 of the Vimentin probed blot), lanes 3 and 5 of the Cleaved Casp-3 probed blot);
- Fig 3B Vimentin panel lane 1 (Mock) and 2 (NT siRNA) appear similar to the Actin panel lane 2 (NT siRNA) and 3 (Vim siRNA);
- Fig 5A PLS-1 blot Ubiquitin panel shows similarity between lanes 1 and 2 as well as lanes 4 and 5 (when adjusted for brightness/contrast);
- Fig 5B, there appear to be repeated areas of similarity within Ubiquitin panel lanes 1, 3, 5;
- There are similar staining patterns in the background of select regions of Fig 6D HDMEC RM, WFA (1μM), LEC STS CM, WFA (1μM) and LEC RM, DMSO panels
- Fig 6B HDMEC and LEC dotplots with WFA (μM/24h) treatment appear to contain similar patterns;
- Fig 7A contains vertical discontinuities after lanes 2 and 4 in the GAPDH blot, and after lane 4 in the Vimentin blot; the background in lane 5 of each panel appear discontinuous with the background in lane 4.
- In Fig 7C the background appears different in lanes 1–4 versus lanes 5–8 of the Vim FL/VDP blot, and there appears to be a vertical discontinuity between lanes 4, 5. In lane 1 of the Activated PARP blot in Fig 7C, there are similarities in the background speck patterns on the left side of the lane.

For concerns regarding flow cytometry data, the authors explain that the data presented in the manuscript are exact replica of the graphs delivered by the institutional core facility. For concerns regarding Western blot panels, the authors note that the background and vertical discontinuities observed could be attributed to a technical artefact. The authors do not agree with the concerns raised and stand by the integrity of their published figures and the validity of the results in the article.

However, in the absence of the data underlying the published figures and in light of the concerns affecting multiple figures and panels depicting different experiments which cast doubt on the reliability of the key findings presented in this study, the *PLOS ONE* Editors retract this article.

GL, AL, and DL did not agree with the retraction. QZ, KH, SW, SB, JL, KT, RL, and MH did not respond.
